# How to Motivate SARS-CoV-2 Convalescents to Receive a Booster Vaccination? Influence on Vaccination Willingness

**DOI:** 10.3390/vaccines10030455

**Published:** 2022-03-16

**Authors:** Elias Kowalski, Andreas Stengel, Axel Schneider, Miriam Goebel-Stengel, Stephan Zipfel, Johanna Graf

**Affiliations:** 1 Department of Psychosomatic Medicine and Psychotherapy, University Hospital Tübingen, 72076 Tübingen, Germany; elias-manuel.kowalski@student.uni-tuebingen.de (E.K.); andreas.stengel@med.uni-tuebingen.de (A.S.); miriam.stengel@helios-gesundheit.de (M.G.-S.); stephan.zipfel@med.uni-tuebingen.de (S.Z.); 2 Health Department Freudenstadt, 72250 Freudenstadt, Germany; axel.schneider@kreis-fds.de; 3 Charité Center for Internal Medicine and Dermatology, Department for Psychosomatic Medicine, Charité-Universitätsmedizin Berlin, Corporate Member of Freie Universität Berlin, Humboldt-Universität zu Berlin and Berlin Institute of Health, 10117 Berlin, Germany; 4 Internal Medicine and Gastroenterology, Helios Klinik Rottweil, 78628 Rottweil, Germany

**Keywords:** associated factors, behavior, COVID-19, infected, mental health, public health, vaccine acceptance, vaccination hesitancy, vaccine intention, vaccine uptake

## Abstract

(1) Background: Booster vaccinations for SARS-CoV-2 convalescents are essential for achieving herd immunity. For the first time, this study examined the influencing factors of vaccination willingness among SARS-CoV-2 infected individuals and identified vaccination-hesitant subgroups. (2) Methods: Individuals with positive SARS-CoV-2 PCR results were recruited by telephone. They completed an online questionnaire during their home isolation in Germany. This questionnaire assessed the vaccination willingness and its influencing factors. (3) Results: 224 home-isolated individuals with acute SARS-CoV-2 infection were included in the study. Vaccination willingness of home-isolated SARS-CoV-2 infected individuals with asymptomatic or moderate course was 54%. The following factors were associated with significantly lower vaccination willingness: younger age, foreign nationality, low income, low trust in vaccination effectiveness, fear of negative vaccination effects, low trust in the governmental pandemic management, low subjective informativeness about SARS-CoV-2, support of conspiracy theories. (4) Conclusions: The vaccination willingness of home-isolated SARS-CoV-2 infected individuals with asymptomatic or moderate symptomatic course was low. Motivational vaccination campaigns should be adapted to individuals with acute SARS-CoV-2 infection and consider the vaccination-hesitant groups. Vaccination education should be demand-driven, low-threshold, begin during the acute infection phase, and be guided for example by the established 5C model (“confidence, complacency, constraints, calculation, collective responsibility”).

## 1. Introduction

On 11 March 2020, the World Health Organization (WHO) classified the spread of the SARS-CoV-2 virus as a pandemic [1]. Since then, the SARS-CoV-2 pandemic has threatened humanity all around the world. By the end of February 2022, more than 435 million people have been infected with the SARS CoV-2 virus, and over 5.9 million people died due to SARS-CoV-2 [2].

Since the beginning of the pandemic, scientists and researchers around the world have been working intensively to develop effective vaccines against SARS-CoV-2. Vaccination is intended to prevent the transmission of the virus, severe courses, and deaths [3]. In this context, 31 December 2020 represents a key turning point in the fight against the pandemic [4]. On this day, the WHO listed the first SARS-CoV-2 vaccine for emergency use [4]. About one year later (end of February 2022), more than 4.9 billion people worldwide were already vaccinated against SARS-CoV-2 [2]. Vaccination has developed as the weapon par excellence of pandemic response. In Germany, 75.4% of the total population was fully vaccinated, and 57.0% received their booster vaccination by the end of February 2022 [5]. However, this is still not enough for a sufficient pandemic control [6]. The Robert Koch Institute (Germany’s national public health institute) has used mathematical modelling to calculate the minimum vaccination rate to achieve herd immunity against SARS-CoV-2 [6]. In July 2021, the Robert Koch Institute postulated that the vaccination rate should be ≥85% for people aged 12–59 years and ≥90% for people aged 60 years and older [6]. If a more transmissible variant occurs (such as omicron), the vaccination rate should be even higher [6]. In any case, there are still too many people who have not been vaccinated against SARS-CoV-2 [6].

Previous efforts to increase vaccination rates have focused mainly on the unvaccinated general population [6]. However, this does not consider the SARS-CoV-2 convalescent, whose proportion is rapidly increasing with the progression of the pandemic. As of the end of February 2022, there were over 11 million people in Germany who had recovered from their SARS-CoV-2 infection, representing 13.8% of the German population [7,8]. Numerous studies indicate that the humoral [9,10,11,12,13,14,15] and cellular [16,17,18,19,20] immune response to SARS-CoV-2 infection provides temporary immunity [9,10,11,12,13,14,15,16,17,18,19,20]. However, natural immunity decreases over time, especially in SARS-CoV-2 infected individuals with moderate course [21]. Reinfections occur, also with different virus variants [22,23,24,25,26]. Reinfected individuals can have high viral loads and thus be very contagious [27]. All these findings support booster vaccinations after recovering from SARS-CoV-2 infection, which lead to successful rebuilding of immunity [28,29].

Therefore, the Standing Committee on Vaccination (STIKO) at the Robert Koch Institute (RKI, Germany’s national Public Health Institute) generally recommends booster vaccinations for all people who have been infected with SARS-CoV-2 [3]. Booster vaccination should be given 3 months after a positive PCR test or 4 weeks after a positive antibody test [3]. In cases of immunodeficiency or exposure to an immune escape variant, SARS-CoV-2 convalescents should be boostered already 4 weeks after a positive PCR test [3]. A further booster vaccination should be given 3 months after the first booster [3].

Booster vaccinations for SARS-CoV-2 convalescents are also recommended in other countries. In the UK, the Health Security Agency (UKHSA) of the Department of Health and Social Care generally recommends booster vaccinations 4 weeks after SARS-CoV-2 infection [30]. In the US, the National Center for Immunization and Respiratory Diseases (NCIRD) of the Centers for Disease Control and Prevention (CDC) generally recommends booster vaccinations for all who have been infected with SARS-CoV-2 [31]. In contrast to the STIKO and the UKHSA, the CDC does not give an exact recommendation on the interval between SARS-CoV-2 infection and booster vaccination [31]. The CDC only declares that SARS-CoV-2 infected individuals should not be vaccinated until they have recovered from the acute illness [31].

According to our knowledge, only two studies have addressed the booster vaccination rate of SARS-CoV-2 convalescents—with contrasting results [32,33]. In the COVIMO study (COVID-19 vaccination rate monitoring in Germany, *n* = 3009), 83% of SARS-CoV-2 convalescents with vaccination recommendation at that time received a booster vaccination after their infection (recorded by random telephone interview) [32]. Thus, SARS-CoV-2 convalescents had a much higher vaccination rate (83%) than the average of the total German population (73.5%) but were still below the recommended vaccination rate (85–90%) [5,6,32]. In contrast, a quota-representative survey (*n* = 5692) from Germany indicates that SARS-CoV-2 convalescents (*n* = 276) had even a significantly lower vaccination willingness compared with those who were not yet infected [33].

Until now, influencing factors and reasons for the low vaccination willingness of SARS-CoV-2 convalescents are still unclear. Therefore, preliminary considerations on this issue need to be based on general behavioral models, psychological models of vaccination behavior [34,35], or refer to pandemic studies of the general population [36,37,38,39,40,41,42,43,44,45,46,47].

Vaccination willingness is deeply connected to behavior and behavioral changes. Two well established behavior models are COM-B [48] and the Fogg Behavior Model [49]. The COM-B is an interactional behavioral system consisting of the following components: “capability” (psychological and physical), “opportunity” (physical and social), and “motivation” (reflective and automatic processes) [48]. In a first step, deficits in these three behavioral components can be identified [48]. In a second step, the deficits can be used to derive interventions for behavioral change, which in a third step are assigned to political categories to enable these interventions [48]. COM-B can contribute to explain vaccination willingness and vaccination behavior.

Further, it is known that preventive vaccination decisions are based on complex psychological processes [34,35]. In 2015, the Strategic Advisory Group of Experts on Immunization (SAGE) of the WHO highlighted the 3C model, “complacency, confidence, and convenience”, substantially influencing vaccination decisions [35]. In 2018, this model was transformed into the now-established 5C model explaining the five psychological antecedents of vaccination: “confidence, complacency, constraints, calculation, and collective responsibility” [34]. For example, the fear of side effects of vaccinations could be presented both in a lower “confidence” in vaccinations and in an intensive “calculation” of the benefits and risks of vaccinations. In general, the 5C model is based on preliminary work by WHO [35] as well as on psychological theories, such as the “theory of planned behavior” [50] or the “health belief model” [51]. The 5C model can help to understand the individual vaccination behavior. After the analysis of this individual vaccination behavior, active support can be provided to address and influence the individual vaccination decision [52]. The following approach addresses the 5C model and can help to increase vaccination willingness: Providing information about the disease and its consequences, educating people about the risks of vaccination, dispelling circulating myths, emphasizing social responsibility, and reducing barriers to vaccination [52].

Since the onset of the SARS-CoV-2 pandemic, numerous systematic reviews examined factors influencing SARS-CoV-2 vaccination willingness in the general population [36,37,38,39,40,41,42,43,44,45,46,47]. Influencing factors are sociodemographic aspects, vaccine-related aspects (such as fear of adverse vaccination reactions), and political beliefs, trust in government, or pre-existing diseases [36,37,38,39,40,41,42,43,44,45,46,47]. Interestingly, SARS-CoV-2 infection itself may also be an influencing factor on vaccination willingness [37]. In a study from the UK (*n* = 762) on the general population, three SARS-CoV-2 convalescents stated that they had already been infected with SARS-CoV-2 and therefore there was no longer need to receive a booster vaccination for SARS-CoV-2 [37]. This indicates that factors influencing the vaccination willingness of SARS-CoV-2 convalescents may differ from those of the general population [33].

However, until now, little was known about the specific influencing factors that motivate the receipt of a booster vaccination in acute SARS-CoV-2 infected individuals. Therefore, this study recorded vaccination willingness of people with active SARS-CoV-2 infection already at the time of the acute infection phase in home isolation. At this point, the experience of the disease and their psychological, cognitive, and emotional processing are still present. Just in this acute phase of SARS-CoV-2 infection, individuals might be more susceptible to motivational vaccination education. There is potential in this target group to make an important contribution to herd immunity and consequently to pandemic control through effective vaccination programs for SARS-CoV-2 convalescents.

This is the first cross-sectional study to investigate factors influencing vaccination willingness in home-isolated individuals with asymptomatic or moderately symptomatic SARS-CoV-2 infection. The overall objective of the study was to understand the vaccination willingness of SARS-CoV-2 infected individuals and to understand how to motivate SARS-CoV-2 convalescents to receive booster vaccinations.

## 2. Materials and Methods

### 2.1. Definitions

The key terms used in this study are defined as follows. This study was conducted on SARS-CoV-2 infected individuals. We use this term referring to people who have acute SARS-CoV-2 infection confirmed by PCR testing and are still in home isolation. This contrasts with SARS-CoV-2 convalescents who have recovered from acute SARS-CoV-2 infection and are no longer in isolation.

In this study, the SARS-CoV-2 vaccination willingness was assessed. This means the acceptance to be vaccinated against SARS-CoV-2 in the future. This contrasts with the SARS-CoV-2 vaccination rate recorded in other studies, which indicates how many people were vaccinated against SARS-CoV-2 in the past.

Furthermore, we recorded SARS-CoV-2 vaccination attitudes in this study. This refers to opinions on other issues related to SARS-CoV-2 vaccination (e.g., thought on mandatory vaccination).

### 2.2. Study Design

This cross-sectional study investigated the vaccination willingness, the vaccination attitudes, and their influencing factors of acutely SARS-CoV-2 infected individuals during home isolation. This study investigated the same study population as Kowalski and colleagues, which was previously used to examine stress and coping depending on psychological burden [53]. Only ambulatory patients with SARS-CoV-2 infection confirmed by PCR diagnosis were included in the study. Patients were in 14-day home isolation at the time of the survey. Recruitment was conducted via the health department of Freudenstadt (Germany). Thus, only patients who were registered citizens in the district of Freudenstadt or who were residing in this district at the time of their SARS-CoV-2 infection were included.

The survey was conducted over an 11-week period between January 2021 and April 2021. During this period, all patients with a positive SARS-CoV-2 PCR result reported to the Freudenstadt Health Department were enrolled (*n* = 838). Patients who were under the age of 18 at the time of the survey (*n* = 142) and patients in nursing homes were excluded (*n* = 48). First, patients were asked by telephone whether they would be interested in participating in the study. Patients with no interest in participation (*n* = 119) and patients with high language barriers (*n* = 51) were excluded.

In case of consent (*n* = 478), an e-mail was sent providing more detailed information about the study and the link to an online questionnaire (EFS survey). Some (*n* = 155) did not access the questionnaire. The online survey was designed to exclude repeated participation (block of IP address). *n* = 99 were excluded from data usage because they did not finish the questionnaire and thus had too many missing values; 224 observations with <7% of missing values were included in the final analyses.

### 2.3. Measures

First, sociodemographic aspects and SARS-CoV-2 associated somatic factors were assessed. Next, SARS-CoV-2 vaccination willingness and attitudes toward vaccination(s) were assessed as central study elements. Furthermore, factors influencing SARS-CoV-2 vaccination willingness and vaccination attitudes were examined: attitudes regarding governmental anti-SARS-CoV-2 measures, subjective informativeness regarding the pandemic, and susceptibility to conspiracy theories. The various measures are further described below.

#### 2.3.1. Sociodemographics

The following sociodemographic aspects were assessed and evaluated: age, gender, nationality, highest educational attainment (for the analysis dichotomized into low educational level (lower or higher secondary education) and high educational level (university entrance qualification or university education)), number of children, number of people in the household, gross income of the household (filled in as free text), and worsening of the financial situation due to the SARS-CoV-2 pandemic.

#### 2.3.2. Somatic Factors

Checklists were used to assess symptoms caused by SARS-CoV-2 infection and risk factors for a severe course. These checklists are based on publications of the Robert Koch Institute (German public health institute) at the time of the survey. SARS-CoV-2 symptoms assessed were, e.g., cough, sniffles, and fever. Assessed risk factors were, e.g., smoking, obesity, and various pre-existing diseases. Sum scores were formed for SARS-CoV-2 symptoms and risk factors. In addition, the subjective severity of physical conditions at the time of the survey and at the time of the worst day of illness was assessed (0 = very bad physical condition to 100 = very good physical condition).

#### 2.3.3. Background of Ad Hoc Scales (Vaccination Attitudes, Anti-Corona Measures, Conspiracy Theories)

Further ad hoc items were used to assess vaccination willingness, vaccination attitudes, attitudes regarding the government’s anti-SARS-CoV-2 measures, subjective informativeness, and susceptibility to conspiracy theories. In the care of SARS-CoV-2 infected patients, the study team was daily confronted with these issues since the start of the pandemic. This extensive expertise enabled the items to be generated. A slider was used to provide individual and intuitive ratings of the items. The scale went from 0 = do not agree at all to 100 = agree completely. For a clearer graphical representation of the data, the scale was subsequently trichotomized (0–33 = do not agree, 34–66 = undecided, 67–100 = agree), similar to other studies [54,55]. The individual topics and the items they contain are presented below. Short forms of the items used in the results and discussion are given in parentheses.

#### 2.3.4. Vaccination Items (Vaccination Willingness and Vaccination Attitudes)

One item was generated to assess the willingness to receive a booster vaccination.

1. “I will get vaccinated against the Corona virus.” (=SARS-CoV-2 vaccination willingness).

Five items were generated to assess attitudes regarding various vaccination topics.

1. “As many people as possible should be vaccinated against the Corona virus in Germany.” (=in favor of a high SARS-CoV-2 vaccination rate)
2. “The Corona pandemic can be defeated with vaccinations.” (=in favor of pandemic defeat with vaccination)
3. “There should be a general mandatory Corona vaccination in Germany.” (=in favor of general mandatory vaccination)
4. “There should be a mandatory Corona vaccination for medical personnel in Germany.” (=in favor of medical mandatory vaccination)
5. “I am afraid of negative effects (e.g., long-term damage) of Corona vaccination.” (=fear of negative vaccination outcomes).

The vaccination item 1 (“in favor of a high SARS-CoV-2 vaccination rate”) and vaccination item 2 (“in favor of pandemic defeat with vaccination”) can be summarized according to their content as pro-vaccination items. The vaccination item 3 (“in favor of general mandatory vaccination”) and vaccination item 4 (“in favor of medical mandatory vaccination”) can be summarized as mandatory vaccination items. The vaccination items were also used to reflect various psychological antecedents of vaccination behavior within the 5C model [34]. For the representation in the 5C model see Table 1.

#### 2.3.5. Attitudes Regarding the Government’s Anti-SARS-CoV-2 Measures

Four ad hoc items assessed attitudes regarding governmental anti-SARS-CoV-2 measures:

1. “I have the feeling that the state and the responsible authorities have the situation regarding the Corona virus under control.” (=situation under control)
2. “I have a feeling that the state and the relevant authorities are concealing the situation regarding the Corona virus.” (=situation concealed)
3. “I think it is reasonable that the federal states each have different regulations. “(=federal regulations)
4. “The government’s ordered measures to contain the pandemic are too strict for me.” (=measures too harsh).

#### 2.3.6. Subjective Informativeness and Susceptibility to Conspiracy Theories

One self-generated item assessed the subjective informativeness:

1. “I feel sufficiently informed about the Corona virus.” (=subjective informativeness).

Four self-generated items were used to assess the susceptibility to conspiracy theories:

1. “The Corona virus was cultivated as a bioweapon in a Chinese laboratory.” (=virus as bioweapon)
2. “The Corona virus was developed by the pharmaceutical industry to earn a lot of money with the help of vaccines.” (=virus developed by pharmaceutical industry)
3. “Corona testing involves the transplantation of microchips.” (=transplantation of microchips during testing)
4. “Corona virus infection is more harmless than the flu.” (=more harmless than the flu).

### 2.4. Statistical Analyses

The statistical analysis of the data was performed using SPSS (version 27). Missing values appeared in 20.1% of the respondents who completed the questionnaire, where mostly only 1–2 items were not answered. Structured analyses of the missing pattern showed a completely random pattern. To replace the missing values, the Fully Conditional Specification Method of Multiple Imputation was used. In total, 20 imputations were performed.

Normal distribution was tested by using the following methods for assessment: comparability of mean and median, skewness, kurtosis, z-standardized skewness, z-standardized kurtosis, Kolmogorov–Smirnov test, Shapiro–Wilk test, histogram, and Q–Q plot. After, detailed evaluation data were found to be not normally distributed. A two-sided Mann–Whitney U test with *p* < 0.05 was performed for significance testing to analyze differences regarding SARS-CoV-2 vaccination willingness and attitudes depending on gender (female/male), foreign nationality (yes/no), educational level (low/high), children (yes/no), and financial pandemic-related losses (yes/no). Furthermore, a two-sided Kruskal–Wallis test with *p* < 0.05 was performed for significance testing to analyze differences regarding SARS-CoV-2 vaccination willingness between young, middle-aged, and old people. The bivariate Spearman’s rho correlation coefficient was calculated to show associations between SARS-CoV-2 vaccination willingness, SARS-CoV-2 vaccination attitudes, further sociodemographic aspects (age, number of household members, income), somatic factors, attitudes toward the government’s regulation, subjective informativeness, and susceptibility to conspiracy theories (two-tailed, *p* < 0.05).

Multivariate analysis by using a binary logistic regression analysis was performed to analyze which factors predicted SARS-CoV-2 vaccination willingness. Vaccination willingness was dichotomized for this purpose (<67 points: no willingness to vaccinate; ≥67 points: willingness to vaccinate). All variables identified as significant in the Mann–Whitney U test, the Kruskal–Wallis test, or Spearman’s rho correlation were included in the regression model as independent variables. A test for multicollinearity was performed by entering all potential model variables into a correlation matrix. Since no variables had correlation values higher than 0.80, no variables had to be excluded, and 17 variables could be included into the model.

## 3. Results

### 3.1. Sample Description

A total of 224 home-isolated SARS-CoV-2 infected individuals was included in the analysis based on full participation in the cross-sectional online survey; 118 (52.7%) respondents were female. Respondents had a median age of 39.0 years; 28 (12.5%) had a migration background; 145 (64.7%) had a low educational level; 145 (64.7%) respondents had children; 122 (54.5%) respondents lived in households with at least three total household members; and 189 (84.4%) lived in municipalities with a maximum of 10,000 inhabitants.

A total of 224 (100%) isolated at home. Respondents completed the survey at a median of 12.0 isolation days. A total of 209 (93.3%) had SARS-CoV-2 associated symptoms (symptomatic course). The most common symptom was headache (63.8%). A total of 101 (45.1%) of the respondents had at least one risk factor for severe SARS-CoV-2 course. The descriptive statistics on sociodemographic aspects and SARS-CoV-2 characteristics can be found in Table 2.

### 3.2. Vaccination Willingness and Attitudes to Further Vaccination Topics

**Figure 1:**
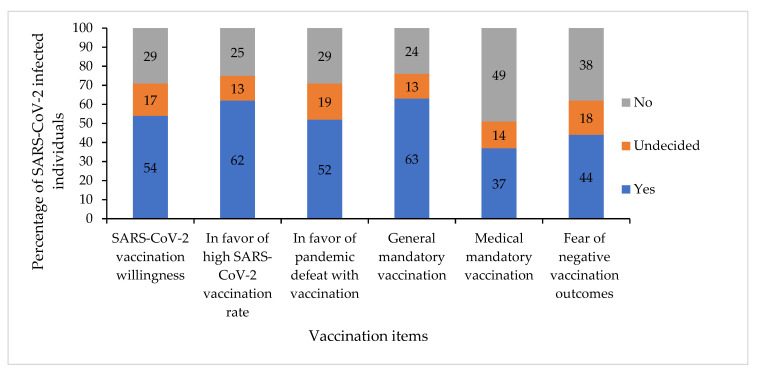
Vaccination attitudes of SARS-CoV-2 infected individuals (*n* = 224).

As a key study element, the vaccination willingness of SARS-CoV-2 infected persons was assessed. Median vaccination willingness was 80.0 scale points (Q1 = 10.0, Q3 = 100.0, range = 0–100). A total of 54% would like to get vaccinated against SARS-CoV-2; 62% said that as many people as possible should be vaccinated against SARS-CoV-2; and 52% believed that SARS-CoV-2 pandemic can be defeated with vaccinations. While only 24% agreed with a general mandatory vaccination against SARS-CoV-2, 27% agreed with a mandatory vaccination for medical personnel; 44% were afraid of negative effects of SARS-CoV-2 vaccination. For a graphical overview of the vaccination attitudes results, see Figure 1.

### 3.3. Vaccination Attitudes Affecting Willingness to Receive Booster Vaccination

The willingness of SARS-CoV-2 infected persons to receive a booster vaccination significantly and with strong effects correlated with other pro-vaccination items (Table 3). The pro-vaccination items and the mandatory vaccination items were positively related with the willingness to get vaccinated against SARS-CoV-2. In contrast, anxiety about adverse effects of vaccination correlated negatively and significantly with vaccination willingness.

### 3.4. Sociodemographic Aspects Affecting Vaccination Attitudes

Several sociodemographic aspects were associated with vaccination willingness (Table 4, Table S1 and Table S2) and vaccination attitudes (Table S3).

Age positively and significantly correlated with vaccination willingness, the pro-vaccination items, and the mandatory vaccination items. Age was negatively related with fear of adverse vaccination outcomes. Interestingly, the Kruskal–Wallis test showed significant differences regarding vaccination willingness between young and middle-aged individuals and between young and old individuals, but not between middle-aged and old individuals (Table S2). Male respondents with SARS-CoV-2 were significantly more likely to agree with a medical mandatory vaccination than female respondents. Female respondents with SARS-CoV-2 significantly more often feared adverse vaccination outcomes compared to male respondents. SARS-CoV-2 infected respondents with local nationality had significantly higher vaccination willingness than respondents with foreign nationality. The other differences in vaccination attitudes regarding gender and nationality were not significant. Respondents with high educational level were significantly more likely to believe that as many as possible should get vaccinated against SARS-CoV-2 than respondents with low educational level (Table S1).

Respondents with children had a significantly higher vaccination willingness than respondents without children (Table S1). There was no significant relationship between number of household members and vaccination attitudes. Income correlated significantly with vaccination willingness and the pro-vaccination items. There were no significant differences in vaccination willingness or attitudes between people with and without financial pandemic-related losses.

### 3.5. Somatic Factors Affecting Vaccination Attitudes

The influence of somatic factors towards vaccination willingness (Table 4) and attitudes was examined (Table S3).

SARS-CoV-2 associated symptoms correlated significantly with the attitude that the pandemic can be defeated by vaccination, while SARS-CoV-2 associated risk factors correlated negatively with this attitude. There was no other significant relationship between somatic factors and vaccination attitudes.

### 3.6. Attitudes toward the Government’s Regulations Affecting Vaccination Attitudes

**Figure 2:**
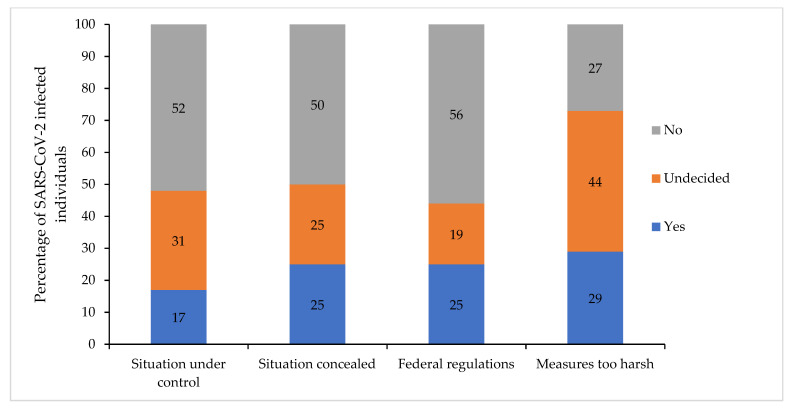
Attitudes toward the government’s regulations (*n* = 224).

For a graphical representation of the government’s regulation data, see Figure 2. Several attitudes towards governmental regulations were associated with vaccination willingness (Table 4) and attitudes (Table S3).

The belief that the government controls the SARS-CoV-2 situation correlated significantly and positively with the vaccination willingness, the pro-vaccination items, and the mandatory vaccination items, and significantly and negatively with the fear of negative vaccination effects (Table 4 and Table S3). Conversely, the belief that the government is concealing the SARS-CoV-2 situation significantly and negatively correlated with the vaccination willingness, the pro-vaccination items, and the mandatory vaccination items, and significantly and positively with the fear of negative vaccination effects (Table 4 and Table S3). Support of federal regulations did not show a significant relationship with vaccination attitudes. The attitude that anti-SARS-CoV-2 measures are too harsh was significantly and negatively correlated with the vaccination willingness and the pro-vaccination items (Table 4 and Table S3).

### 3.7. Subjective Informativeness and Susceptibility to Conspiracy Theories Affecting Vaccination Attitudes

**Figure 3:**
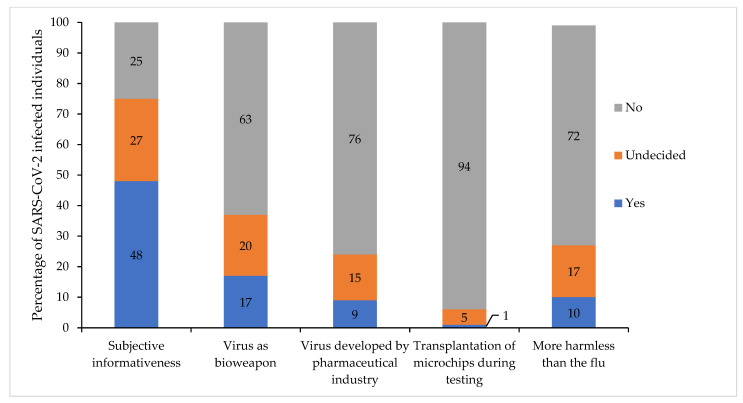
Subjective informativeness and susceptibility to conspiracy theories (*n* = 224).

A graphical representation of the subjective informativeness and the susceptibility to conspiracy theories data is shown in Figure 3). Subjective informativeness and susceptibility to several conspiracy theories were associated with vaccination willingness (Table 4) vaccination attitudes (Table S3).

Subjective informativeness significantly and positively correlated with vaccination willingness, pro-vaccination items, mandatory vaccination items, and significantly and negatively with the fear of negative vaccination effects (Table 4 and Table S3). In summary, acceptance of various conspiracy theories was significantly and negatively correlated with vaccination willingness (Table 4).

### 3.8. Prediction of SARS-CoV-2 Vaccination Willingness

The binary logistic regression analysis to identify predictors of SARS-CoV-2 vaccination willingness contained the 17 variables that showed a significant correlation with SARS-CoV-2 vaccination willingness. The model considering all variables was significant (*x*^2^ = 185.28, *p* < 0.001) and thus was able to distinguish between vaccine willing and not vaccine willing respondents. The model explained 56% (Cox & Snell *R*^2^ = 0.56), or 74% (Nagelkerke’s *R*^2^ = 0.74) of the variance; 89.3% of the cases could be predicted correctly. Nevertheless, there were only a few independent significant factors related to SARS-CoV-2 vaccination willingness. Only the vaccination attitudes “In favor of a high SARS-CoV-2 vaccination rate”, “In favor of pandemic defeat with vaccination”, and “Fear of negative vaccination outcomes” independently and significantly predicted SARS-CoV-2 vaccination willingness (Table 5).

## 4. Discussion

This is the first cross-sectional study that examined SARS-CoV-2 vaccination willingness and its influencing factors in individuals with acute SARS-CoV-2 infection. Vaccination willingness of the 224 home-isolated respondents with SARS-CoV-2 was only 54%. In the bivariate correlation analyses, the following factors were identified as affecting the vaccination attitudes of SARS-CoV-2 infected individuals: sociodemographic aspects, somatic factors, attitudes towards vaccination, attitudes towards governmental regulations, subjective informativeness, and susceptibility to conspiracy theories. In the binary logistic regression analysis, only three independent significant predictors remained (“In favor of a high SARS-CoV-2 vaccination rate”, “In favor of pandemic defeat with vaccination”, and “Fear of negative vaccination outcomes”). In the following, we discuss the prediction of SARS-CoV-2 vaccination willingness and further factors associated with SARS-CoV-2 vaccination using the 5C model on psychological antecedents of vaccination decisions [34]. Further, we frame our study results in the behavioral model COM-B [48].

### 4.1. Prediction of SARS-CoV-2 Vaccination Willingness

In the binary logistic regression model to predict SARS-CoV-2 vaccination willingness, there were only three independent significant predictors related to SARS-CoV-2 vaccination (“In favor of a high SARS-CoV-2 vaccination rate”, “In favor of pandemic defeat with vaccination”, and “Fear of negative vaccination outcomes”). Furthermore, the regression coefficients and odds ratios were all quite low. Nevertheless, a few conclusions can be drawn from the model.

The strongest predictor of SARS-CoV-2 vaccination willingness was the belief that the SARS-CoV-2 pandemic could be defeated by vaccination. The second strongest predictor of SARS-CoV-2 vaccination willingness was the belief that as many people as possible should be vaccinated against SARS-CoV-2. Thus, a higher “confidence” in SARS-CoV-2 vaccinations and their effectiveness led to an increased SARS-CoV-2 vaccination willingness. Lower “confidence” in the safety of vaccinations represented by a higher fear of negative vaccination effects resulted in lower SARS-CoV-2 vaccination willingness. This might be reflected in a “calculation” process between the benefits and risks of vaccination. Infected individuals may fear that the vaccination will cause symptoms similar to their SARS-CoV-2 infection. On the other hand, the experience of an asymptomatic or moderate SARS-CoV-2 course may reduce the fear of vaccination side effects. Studies on the general population confirm that fear of side effects, or a lack of confidence in vaccine safety, results in a lower willingness to vaccinate [36,37,38,40,42,43,46,47]. Furthermore, a high level of trust in the vaccination effectiveness positively influences the willingness to vaccinate in the general population [36,38,40,56].

Thus, the results of the regression model imply that confidence building in SARS-CoV-2 vaccinations is a significant factor in increasing SARS-CoV-2 vaccination willingness among SARS-CoV-2 infected individuals. Therefore, it is important that public campaigns address the safety of vaccination, so that a high level of confidence in the vaccine is created.

### 4.2. Further Factors Associated with SARS-CoV-2 Vaccination Willingness

The following discussion about the results of the bivariate correlations should be taken with caution, as they were not confirmed as independent factors in the binary logistic regression. Nevertheless, they provide important indications that are also consistent with the results of previous studies on the general population. These indications should be included and explored in further studies with SARS-CoV-2 infected individuals.

In this study, lower age, lower income, and foreign nationality were associated with lower vaccination willingness in SARS-CoV-2 infected individuals. This has already been shown in reviews with studies on the general population [36,38,39,42,43,44,46,47]. Presumably, governmental vaccination campaigns do not effectively reach these groups. Structural barriers to getting vaccinated may be too big (high “constraints”).

Interestingly, health status, number of SARS-CoV-2 symptoms, and risk factors did not correlate with vaccination willingness in this study. In contrast, risk groups in the general population see their risk for a severe course as a reason for vaccination [43]. It seems that high-risk SARS-CoV-2 infected persons downplay their risk after an infection and thus expose themselves to the risk of re-infection without booster vaccination. This issue should be addressed in further vaccination campaigns.

Confidence in governmental pandemic response correlated positively with vaccination willingness among SARS-CoV-2 infected individuals in this study. These correlations were also shown in the general population [36,37,38,40,42,43,47]. Governments should therefore base their decisions regarding the pandemic response strictly on scientific evidence [57]. Policy decisions should be science-driven, so they can be communicated to the public in a transparent, coherent, understandable, clear, and credible manner overcoming the challenges of the “infodemic” [57]. This may provide trust in scientifically developed vaccines and government-initiated vaccination campaigns (“confidence”) [57].

High subjective informativeness about the SARS-CoV-2 pandemic and low susceptibility to various conspiracy theories were associated with increased SARS-CoV-2 vaccination willingness in this study. Vaccination campaigns should use transparent information and communication to encourage reflective “calculation” processes. The “calculation” process can then be used to increase “confidence” in vaccination and “collective responsibility”.

### 4.3. SARS-CoV-2 Vaccination Willingness in the Context of the COM-B Behavioral Model and Behavior Change Interventions

According to COM-B, “capability”, “opportunity”, and “motivation” are the three conditions for behavior [48]. They all might play a central role in vaccination willingness and its influencing factors of SARS-CoV-2 infected individuals. It is likely that in principle most respondents will have the physical ability to receive a SARS-CoV-2 vaccination after recovery, because there are only very few contraindications to SARS-CoV-2 vaccination [30]. Nevertheless, SARS-CoV-2 vaccinations should be given only after recovery is fully completed [3,30,31]. Thus, the optimal physical ability to receive the SARS-CoV-2 booster vaccination is achieved only over time. This might negatively influence vaccination willingness of individuals with acute SARS-CoV-2 infection.

Psychological capability might play a central role in vaccination decisions of SARS-CoV-2 infected individuals. This includes, for example, required knowledge [48]. This study showed that people with low subjective informativeness or high susceptibility to conspiracy theories had lower vaccination willingness. This issue can be addressed through education and imparting of knowledge [48]. Policy makers should keep this in mind in their communication strategies and vaccination campaigns.

Opportunity establishes a baseline for vaccination willingness among SARS-CoV-2 infected individuals. Governments should therefore improve opportunity through confidence-building measures [48].

We expected motivational aspects to play a major role in the context of vaccination willingness of SARS-CoV-2 infected individuals. In this study, fear of negative vaccination outcomes negatively influenced vaccination willingness. Both reflective and automatic processes could influence this factor. It is understandable that a newly researched vaccination is impulsively approached with skepticism and negative emotions such as fear. Reflective, evaluative processes may also have an impact on the motivational process [48]. The reflective and automatic motivational processes can be addressed with counter-impulses and positive feelings related to the behavioral goal [48]. For example, confidence in vaccinations, gratitude about the expected vaccination protection, and social responsibility could be strengthened.

### 4.4. Recommendations for Clinical Practice

Vaccination campaigns should be established that address the specific situation of SARS-CoV-2 infected individuals [33]. In this study, high confidence in the safety of vaccination and high confidence in the effectiveness of vaccination were the strongest predictors of SARS-CoV-2 vaccination willingness.

Vaccination campaigns should be based on the 5C model, COM-B, and the Fogg Behavior Model to address vaccination behavior at a psychological-interventional level [34,35,48,49,52]. For example, this study showed that fear of adverse vaccination effects (such as long-term consequences) was associated with lower vaccination willingness. In the context of the 5C model, this can be explained by reduced “confidence” [34,35]. Reduced trust can be countered with eye-level education that actively addresses misinformation [52]. Comprehensive education, including vaccine side effects and their association with preexisting conditions, is important [58]. In our view, the psychological processes of “complacency” [34,35] may play a role in the specific situation of SARS-CoV-2 infected individuals. Recently recovered individuals may initially rely on the immune protection acquired through SARS-CoV-2 infection [33,37]. Some may feel safe from reinfection and thus may not get vaccinated against SARS-CoV-2 infection [33,37]. As part of the special vaccination education for SARS-CoV-2 infected persons, health departments and family doctors should clarify that immune protection declines after infection, and reinfection can occur [37]. For the other “Cs” of the 5C model, literature also provides guidance on motivational vaccination education [52]. For improving vaccination willingness of SARS-CoV-2 infected individuals, health departments and family doctors may provide information about the disease and its consequences, educate about the risks of vaccination, dispel circulating myths, emphasize social responsibility, and reduce barriers to vaccination [52]. Digital interventions represent important opportunities for vaccination education campaigns [37] and may be particularly useful during the acute phase of SARS-CoV-2 infection. To achieve behavioral change toward vaccination willingness, deficits should be identified and addressed in the COM-B and Fogg Behavioral Model [48,49].

### 4.5. Limitations

This is the first study addressing vaccination willingness and its influencing factors of SARS-CoV-2 infected individuals. This study is a cross-sectional online survey. Due to the study design, there are several limitations.

The online survey questions were self-completed, which may be associated with bias. All question scales were self-generated and were not validated in advance by a separate study. The questions on vaccination topics remained general and did not address symptom severity. Further studies should better adapt the questions to the specific situation of SARS-CoV-2 infected individuals and better address the specific motivational aspects. Somatic symptoms were assessed based on the Robert Koch Institute (Germany’s national Public Health Institute), but no clinical diagnoses were made. For this purpose, a personal clinical anamnesis would have been necessary.

The survey was regionally limited to a very rural area in Germany. People in rural areas could potentially have lower vaccination willingness [38,43], but this influencing factor is still controversial [42].

The surveys were completed between January and April 2021. On January 29th, 2021, the Standing Committee on Vaccination at the Robert Koch Institute for the first time published a vaccination recommendation for people who underwent SARS-CoV-2 infection confirmed by laboratory diagnostics [59]. At the end of April 2021, only 27.9% of the German population had been vaccinated at least once due to the vaccine deficiency and the associated limited access to vaccinations [5]. Thus, the SARS-CoV-2 vaccines were not yet as established. The timing of the survey could thus lead to an underestimation of vaccination willingness.

The timing of the survey during the acute infection, with all its psychological, cognitive, and emotional processes, could have influenced the survey response. SARS-CoV-2 vaccination willingness may change in convalescents compared to acutely infected individuals as the individual risk of reinfection increases after the infection. People in nursing homes had to be excluded from the outset due to the threatening pandemic situation in the facilities and possible cognitive impairments. Age correlated significantly with vaccination willingness, so due to this exclusion criterion, vaccination willingness might be underestimated.

People with high language barrier had to be excluded. Non-German respondents had a significantly lower vaccination willingness, so this exclusion criterion could lead to an overestimation of vaccination willingness.

In this study, there were only SARS-CoV-2 infected individuals with a moderate course (home isolation) who did not require hospitalization. In this study, although somatic factors did not influence vaccination willingness, the psychological processes around vaccination behavior might still differ among SARS-CoV-2 infected individuals with a severe course. SARS-CoV-2 infected individuals with a severe course might have a higher vaccination willingness because they want to avoid reinfection. As this study only involved a subgroup of SARS-CoV-2 infected individuals, vaccination willingness cannot be generalized.

We strongly suggest that further representative studies on vaccination willingness and its influencing factors of SARS-CoV-2 infected individuals be conducted. Further studies should be supra-regional, longitudinal, and prospective. Further research investigation should also include children, people in nursing homes, people with language barriers, and SARS-CoV-2 infected individuals with severe courses (e.g., hospitalized participants). Studies on the development, establishment, and effectiveness of psychological-interventional vaccination education specifically targeting the situation of SARS-CoV-2 infected persons should be conducted.

## 5. Conclusions

1. For the first time, it has been shown that the vaccination willingness of home-isolated people with acute SARS-CoV-2 infection with asymptomatic or moderate symptomatic course is only 54%.
2. High confidence in the safety of vaccination and high confidence in the effectiveness of vaccination were the strongest predictors of SARS-CoV-2 vaccination willingness.
3. Therefore, broad vaccination education should already take place in the phase of acute SARS-CoV-2 infection. Due to the current psychological processing, affected persons may then be particularly susceptible to motivational vaccination education.
4. Motivational vaccination education should be based on the 5C model (psychological reasons for vaccination behavior): “confidence, complacency, constraints, calculation, collective responsibility” [34]. Vaccination education should be demand-driven, low-threshold, and adapted to the specific situation of SARS-CoV-2 infected individuals, considering the vaccine hesitant subgroups mentioned above.
5. Individual interventions to increase vaccination willingness can be derived from the behavioral models COM-B [48].

## Figures and Tables

**Table 1:** Vaccination items and their representation in the 5C model.

Vaccination Items	Confidence	Complacency	Constraints	Calculation	Collective Responsibility
“As many people as possible should be vaccinated against the coronavirus in Germany.”	X	X			X
“The corona pandemic can be defeated with vaccinations.”	X	X			
“There should be a general mandatory Corona vaccination in Germany.”	X	X	X		X
“There should be a mandatory Corona vaccination for medical personnel in Germany.”	X	X	X		X
“I am afraid of negative effects (e.g., long-term damage) of Corona vaccination.”	X			X	

**Table 2:** Sample description (*n* = 224).

Sociodemographics	Total (%) Median (Q1–Q3)
Age, median	39.0 (28.0–52.0)
Age	
Young (18–29)	64 (28.6%)
Middle-aged (30–59)	135 (60.3%)
Old (≥60)	25 (11.2%)
Gender	
Female	118 (52.7%)
Male	106 (47.3%)
Diverse	0 (0.0%)
Foreign nationality	
Yes	28 (12.5%)
No	196 (87.5%)
Highest educational level	
Low (lower or higher secondary education)	145 (64.7%)
High (university entrance qualification or university education)	79 (35.3%)
Gross income of the household (€ per year), median	60,000 (3950–90,000)
Financial pandemic-related losses	
Yes	109 (48.7%)
No	115 (51.3%)
Children	
Yes	145 (64.7%)
No	79 (35.3%)
Total household members	
Living alone	28 (12.5%)
Living in pairs	74 (33.0%)
Living at least in threes	122 (54.5%)
Size of municipalities	
≤10,000	189 (84.4%)
>10,000	35 (15.6%)
**SARS-CoV-2 characteristics**	
Place of isolation	
At home	224 (100%)
Completion of the survey on isolation day, median	12.0 (10.0–14.0)
SARS-CoV-2 associated symptoms	
Yes (symptomatic course)	209 (93.3%)
No (asymptomatic course)	15 (6.7%)
Most common SARS-CoV-2 symptoms	
headache	143 (63.8%)
sniffles	141 (62.9%)
cough	138 (61.6%)
symptoms of fatigue	129 (57.6%)
body aches	108 (48.2%)
disturbance of the sense of smell	101 (45.1%)
Physical health (0 = very bad, 100 = very good)	
Health at the time of the survey	82.0 (61.0–93.8)
Health at the previous disease peak	48.0 (28.0–70.0)
SARS-CoV-2 associated risk factors	
Yes	101 (45.1%)
No	123 (54.9%)

**Table 3:** Correlations of vaccination attitudes (*n* = 224).

		SARS-CoV-2 Vaccination Willingness	In Favor of a High SARS-CoV-2 Vaccination Rate	In Favor of Pandemic Defeat with Vaccination	In Favor of General Mandatory Vaccination	In Favor of Medical Mandatory Vaccination
In favor of a high SARS-CoV-2 vaccination rate	*r*	0.727				
	*p*	<0.001 ***				
In favor of pandemic defeat with vaccination	*r*	0.759	0.731			
	*p*	<0.001 ***	<0.001 ***			
In favor of general mandatory vaccination	*r*	0.547	0.529	0.527		
	*p*	<0.001 ***	<0.001 ***	<0.001 ***		
In favor of medical mandatory vaccination	*r*	0.464	0.498	0.419	0.739	
	*p*	<0.001 ***	<0.001 ***	<0.001 ***	<0.001 ***	
Fear of negative vaccination outcomes	*r*	−0.463	−0.416	−0.366	−0.371	−0.323
	*p*	<0.001 ***	<0.001 ***	<0.001 ***	<0.001 ***	<0.001 ***

*r* = Spearman’s rho correlation coefficient. *p* = *p*-value. *** *p* < 0.001. Significant correlations are displayed in bold.

**Table 4:** Correlations of sociodemographic aspects, attitudes toward the government’s regulations, subjective informativeness, and susceptibility to conspiracy theories with SARS-CoV-2 vaccination willingness (*n* = 224).

Items		Correlation with SARS-CoV-2 Vaccination Willingness
**Sociodemographic aspects**		
Age	*r*	0.308
	*p*	<0.001 ***
Number of household members	*r*	−0.022
	*p*	0.746
Income (free text)	*r*	0.226
	*p*	0.001 **
**Somatic factors**		
Current health	*r*	−0.002
	*p*	0.972
Worst health	*r*	−0.007
	*p*	0.917
Symptoms	*r*	0.056
	*p*	0.407
Risk factors	*r*	−0.062
	*p*	0.359
**Attitudes toward governmental regulations**		
Situation under control	*r*	0.278
	*p*	<0.001 ***
Situation concealed	*r*	−0.288
	*p*	<0.001 ***
Federal regulations	*r*	0.096
	*p*	0.152
Measures too harsh	*r*	−0.222
	*p*	0.001 **
**Subjective informativeness and susceptibility to conspiracy theories**		
Subjective informativeness	*r*	0.315
	*p*	<0.001 ***
Virus as bioweapon	*r*	−0.136
	*p*	0.041 *
Virus developed by pharmaceutical industry	*r*	−0.220
	*p*	0.001 **
Transplantation of microchips during testing	*r*	−0.175
	*p*	0.009 **
More harmless than the flu	*r*	−0.240
	*p*	<0.001 ***

*r* = Spearman’s rho correlation coefficient. *p* = *p*-value. * *p* < 0.05, ** *p* < 0.01, *** *p* < 0.001. Significant correlations are displayed in bold.

**Table 5:** Predictors for vaccination willingness, determined by a binary logistic regression analysis (*n* = 224).

	Regression Coefficient	Standard Error	Significance	Odds Ratio	95% Confidence INTERVAL for Exp(B)	
Explanatory Variable	B	SE	*p*	Exp(B)	Lower Bound	Upper Bound
**Sociodemographic aspects**						
Age			0.800			
Age ^a^	−0.071	0.724	0.922	0.932	0.225	3.854
Age ^b^	−0.648	1.084	0.550	.523	0.063	4.377
Nationality ^c^	1.184	0.839	0.158	3.269	0.631	16.941
Income	0.000	0.000	0.755	1.000	1.000	1.000
Children ^d^	1.116	0.647	0.084	3.054	0.860	10.851
**Vaccination attitudes**						
In favor of a high SARS-CoV-2 vaccination rate	0.025	0.010	0.015 *	1.025	1.005	1.046
In favor of pandemic defeat with vaccination	0.048	0.011	<0.001 ***	1.049	1.027	1.071
In favor of general mandatory vaccination	0.010	0.009	0.244	1.010	0.993	1.028
In favor of medical mandatory vaccination	0.005	0.008	0.569	1.005	0.989	1.021
Fear of negative vaccination outcomes	−0.017	0.008	0.031 *	0.983	0.968	0.998
**Attitudes toward the government’s regulations**						
Situation under control	−0.003	0.011	0.793	0.997	0.977	1.018
Situation concealed	0.011	0.009	0.209	1.011	0.994	1.029
Measures too harsh	−0.002	0.010	0.819	0.998	0.978	1.018
**Subjective informativeness and conspiracy theories**						
Subjective informativeness	0.010	0.010	0.322	1.010	0.990	1.030
Virus as bioweapon	−0.011	0.009	0.220	0.989	0.972	1.007
Virus developed by pharmaceutical industry	0.002	0.012	0.839	1.002	0.980	1.025
Transplantation of microchips during testing	−0.015	0.018	0.415	0.986	0.952	1.021
More harmless than the flu	0.005	0.009	0.610	1.005	0.986	1.024

Omnibus test of model coefficients: *x*^2^= 185.28, df = 18, *p* < 0.001. Cox & Snell *R*^2^ = 0.56; Nagelkerke’s *R*^2^ = 0.75. Analysis of the classification results: 89.3% of cases were correctly predicted/classified (vaccination willingness: 91.8%, no vaccination willingness: 86.3%). ^a^ Reference category: people between 30 and 59 years. ^b^ Reference category: people ≥ 60 years. ^c^ Reference category: local nationality. ^d^ Reference category: Children. * *p* < 0.05, *** *p* <0.001. Significant predictors are shown in bold.

## Data Availability

The corresponding author will make the raw data available without any restrictions upon request. Please free to use the contact details above.

## Supplementary Materials

The following supporting information can be downloaded at https://www.mdpi.com/article/10.3390/vaccines10030455/s1, Table S1: Differences in vaccination attitudes regarding sociodemographic aspects; Table S2: Differences in vaccination willingness regarding age; Table S3: Differences in vaccination attitudes regarding sociodemographic aspects, somatic factors, attitudes toward governmental regulations, subjective informativeness, and susceptibility to conspiracy theories.
