# Supplementary material for: How to Motivate SARS-CoV-2 Convalescents to Receive a Booster Vaccination? Influence on Vaccination Willingness
Source: Vaccines (Basel). 2022 Mar 16;10(3):455. doi: 10.3390/vaccines10030455 (PMC8953711; doi:10.3390/vaccines10030455)
Supplement: Supplementary file 1 [file vaccines-10-00455-s001.zip › vaccines-1596587-supplementary.pdf]

vaccines MDPI

Supplementary Materials

How to Motivate SARS-CoV-2 Convalescents to Receive a Booster Vaccination? Influence on Vaccination Willingness

Elias Kowalski [1,2], Andreas Stengel [1,3], Axel Schneider [2], Miriam Goebel-Stengel [1,4], Stepan Zipfel [1], and Johanna Graf [1,*]

1 Department of Psychosomatic Medicine and Psychotherapy, University Hospital Tübingen, 72076 Tübingen, Germany
2 Health Department Freudenstadt, 72250 Freudenstadt, Germany
3 Charité Center for Internal Medicine and Dermatology, Department for Psychosomatic Medicine, Charité-Universitätsmedizin Berlin, Corporate Member of Freie Universität Berlin, Humboldt-Universität zu Berlin and Berlin Institute of Health, 10117 Berlin, Germany
4 Internal Medicine and Gastroenterology, Helios Klinik Rottweil, 78628 Rottweil, Germany
* Correspondence: johanna.graf@med.uni-tuebingen.de ; Tel.: +49 7071 29-86719

Table S1. Differences in vaccination attitudes regarding sociodemographic aspects (n = 224).

	SARS-CoV-2 vaccination willingness	In favor of a high SARS-CoV-2 vaccination rate	In favor of pandemic defeat with vaccination	In favor of general mandatory vaccination	In favor of medical mandatory vaccination	Fear of adverse vaccination outcomes
			**Gender**			
U	6121.5	5725.5	5712.0	5691.0	4897.5	5230.5
Z	−0.282	−1.131	−1.129	−1.263	−2.912	−2.128
Mdn (female)	80.0	81.0	73.5	0.0	10.0	69.5
Mdn (male)	80.0	98.0	65.5	2.0	52.5	50.0
p	0.778	0.258	0.259	0.207	**0.004 ****	**0.033 ***
			**Nationality**			
U	2563.5	2792.5	3073.0	3259.5	3071.0	2957.5
Z	−2.178	−1.515	−0.672	−0.148	−0.698	−1.001
Mdn (local nationality)	81.0	91.0	70.0	0.0	41.0	50.0
Mdn (foreign background)	50.0	60.0	54.0	0.0	30.0	52.0
p	**0.029 ***	0.130	0.502	0.882	0.485	0.317
			**Educational level**			
U	5362.5	4839.0	5425.5	5316.5	5174.0	4941.5
Z	−0.812	−1.987	−0.657	−0.963	−1.241	−1.708
Mdn (low education)	73.0	81.0	66.0	0.0	27.0	60.0
Mdn (high education)	83.0	99.0	70.0	10.0	50.0	32.0
p	0.417	**0.047 ***	0.511	0.335	0.214	0.088
			**Children**			
U	4835.0	4897.0	4364.5	5193.0	5140.5	5482.0
Z	−1.986	−1.857	−2.966	−1.253	−1.317	−0.533
Mdn (no children)	52.0	77.0	50.0	0.0	20.0	57.0
Mdn (children)	83.0	95.0	78.0	4.0	50.0	50.0
p	**0.047 ***	0.063	**0.003 ****	0.210	0.188	0.594
			**Financial pandemic-related losses**			
U	6053.5	5756.5	5463.0	6091.5	6131.0	6142.5
Z	−0.455	−1.092	−1.674	−0.394	−0.293	−0.260
Mdn (no loss)	81.0	93.0	71.0	0.0	25.0	50.0
Mdn (loss)	71.0	81.0	61.0	0.0	50.0	52.0
p	0.649	0.275	0.094	0.693	0.770	0.795

*Statistics performed with Mann-Whitney U test. U = Mann-Whitney U. Z = z-value. p = p-value. * $p < 0.05$, ** $p < 0.01$. Bold values are significant.*

Table S2. Differences in vaccination willingness regarding age (n = 224).

	Standard test statistics	Standard error	Significance (p)
Young-Middle	−3.545	9.538	<0.001 ***
Young-Old	−3.233	14.823	0.001 **
Middle-Old	−1.031	0.302	0.907

*Statistics performed with Kruskal-Wallis test. p = p-value. ** p < 0.01, *** p < 0.001. Bold values are significant.*

Table S3. Differences in vaccination attitudes regarding sociodemographic aspects, somatic factors, attitudes toward governmental regulations, subjective informativeness, and susceptibility to conspiracy theories (n = 224).

		In favor of a high SARS-CoV-2 vaccination rate	In favor of pandemic defeat with vaccination	In favor of general mandatory vaccination	In favor of medical mandatory vaccination	Fear of adverse vaccination outcomes
**Sociodemographic aspects**						
Age	*r*	0.307	0.351	0.158	0.150	−0.157
	p	**<0.001 *****	**<0.001 *****	**0.018 ***	**0.024 ***	**0.019 ***
Number of children	*r*	0.076	0.168	0.066	0.047	−0.016
	p	0.255	**0.012 ***	0.324	0.481	0.817
Number of household members	*r*	−0.017	0.020	0.046	0.057	0.068
	p	0.796	0.764	0.497	0.398	0.310
Income (free text)	*r*	0.289	0.203	0.112	0.088	−0.187
	p	**<0.001 *****	**0.002 ****	0.094	0.188	**0.005 ****
Financial pandemic-related losses	*r*	−0.137	−0.171	−0.042	0.015	0.036
	p	**0.040 ***	**0.010 ***	0.531	0.828	0.597
**Somatic Factors**						
Current health	*r*	0.035	−0.007	0.040	−0.009	−0.113
	p	0.606	0.916	0.548	0.894	0.091
Worst health	*r*	0.082	0.042	0.060	0.052	−0.061
	p	0.222	0.535	0.374	0.439	0.365
Symptoms	*r*	−0.026	0.137	−0.016	−0.057	0.130
	p	0.700	**0.041 ***	0.817	0.393	0.051
Risk factors	*r*	−0.024	−0.138	−0.107	0.002	0.089
	p	0.723	**0.039 ***	0.109	0.978	0.182
**Attitudes toward government´s reguluations**						
Situation under control	*r*	0.245	0.292	0.191	0.178	−0.256
	p	**<0.001 *****	**<0.001 *****	**0.004 ****	**0.008 ****	**<0.001 *****
Situation concealed	*r*	−0.432	−0.376	−0.254	−0.260	0.283
	p	**<0.001 *****	**<0.001 *****	**<0.001 *****	**<0.001 *****	**<0.001 *****
Federal regulations	*r*	0.080	0.077	0.052	0.082	−0.047
	p	0.236	0.253	0.438	0.221	0.484
Measures too harsh	*r*	−0.160	−0.212	−0.098	−0.066	0.037
	p	**0.017 ***	**0.001 ****	0.145	0.325	0.583
**Subjective informativeness and susceptibilitiy to conspiracy theories**						
Subjective informativeness	*r*	0.384	0.311	0.226	0.220	−0.317
	p	**<0.001 *****	**<0.001 *****	**0.001 ****	**0.001 ****	**<0.001 *****
Virus as bioweapon	*r*	−0.192	−0.077	−0.026	−0.066	0.344
	p	**0.004 ****	0.253	0.701	0.328	**<0.001 *****
Virus developed by pharmaceutical industry	*r*	−0.277	−0.251	−0.027	−0.060	−0.145
	p	**<0.001 *****	**<0.001 *****	0.684	0.373	**0.030 ***
Transplantation of microchips during testing	*r*	−0.244	−0.213	−0.066	−0.074	0.123
	p	**<0.001 *****	**0.001 ****	0.328	0.270	0.067
More harmless than the flu	*r*	−0.275	−0.278	−0.209	−0.102	0.096
	p	**<0.001 *****	**<0.001 *****	**0.002 ****	0.127	0.152

*r = Spearman´s rho correlation coefficient. p = p-value. * p < 0.05, ** p < 0.01, *** p < 0.001. Bold values are significant.*
